# Efficacy of pharmacotherapy and non-pharmacotherapy of Alzheimer dementia: A protocol for systematic review and meta-analysis

**DOI:** 10.1097/MD.0000000000032382

**Published:** 2022-12-16

**Authors:** Chitima Boongird, Worapong Tearneukit, Wannisa Wongpipathpong, Gareth J McKay, Ammarin Thakkinstian

**Affiliations:** a Chakri Naruebodindra Medical Institute, Faculty of Medicine Ramathibodi Hospital, Mahidol University, Samut Prakan, Thailand; b Neuropsychiatry Unit, Somdet Chao Praya Institute of Psychiatry, Thailand; c Centre for Public Health, Queen’s University Belfast, Belfast, United Kingdom; d Department of Clinical Epidemiology and Biostatistics, Faculty of Medicine Ramathibodi Hospital, Mahidol University, Bangkok, Thailand.

**Keywords:** Alzheimer dementia, network meta-analysis, non-pharmacotherapy, pharmacotherapy

## Abstract

Alzheimer disease (AD) is the most common cause of dementia worldwide. Despite the publication of numerous systematic reviews and meta-analyses that have summarized the evidence associated with pharmacotherapies (PTs) and non-pharmacotherapies (NPTs) for the wide range of interventions available for AD treatment, their comparative safety and efficacy remains insufficiently defined.

**Methods:**

Systematic reviews of randomized controlled trials (RCTs) will be selected according to the following criteria: conducted in elderly patients aged 60 years or older with AD living in community or institutionalized settings, applied pairwise meta-analysis (PMA) or network meta-analysis (NMA) approaches providing pooled relative treatment effects for at least 1 pair of PTs or NPTs, and providing at least 1 of the following outcomes for patients/caregivers: cognitive, functional status, behavior, quality of life (QoL), and caregiver stress or burden. All article screening, data extraction, and risk of bias assessment will be completed independently by 2 reviewers. Relative treatment rankings will be reported with mean ranks and surface under the cumulative ranking curves.

**Results and Conclusion:**

We will determine the most efficacious treatment strategies for AD patients from the most highly ranked treatments. These results will help to guide clinical decision-making and improve patient care.

## 1. Introduction

Alzheimer disease (AD) is an age-related neurodegenerative condition with increased prevalence in older populations. The World Health Organization (WHO) estimated more than 50 million people were living with dementia, with approximately 60 percent originating from low and middle-income countries.^[1]^ The economic burden of AD is anticipated to increase along with rising prevalence.^[1]^ In Thailand, almost 600,000 individuals were reported with dementia in 2015 with expectations this will exceed 1 million by 2030.^[2]^ The familial, societal and economic burdens associated with AD are substantial with a recent survey indicating 86% of dementia caregivers in Thailand were provided informally from the same household source with the bulk of associated care costs characterized as indirect due to lost productivity of unpaid caregivers.^[2]^

Many systematic reviews, including several Cochrane Collaborations, have used pairwise meta-analysis (PMA) and network meta-analysis (NMA) approaches,^[3–8]^ to identify pharmacological treatments (PT) that reduce the rate of cognitive decline in older adults with early-stage dementia. Several reviews^[5,8,9]^ recommended the use of acetylcholinesterase inhibitors (AChEIs) such as donepezil, rivastigmine, and galantamine, in addition to memantine, an uncompetitive antagonist of N-methyl-D-aspartate (NMDA) glutamate receptors, for the treatment of moderate to severe dementia. Separately, herbal remedies such as huperzine A,^[10]^ ginkgo biloba leaf (EGb761),^[11]^ and traditional Chinese medicines^[12]^ have been shown to provide clinical benefits on cognitive function similar to that observed following treatment with AChEIs. However, many of these PTs have been associated with adverse effects and greater risk of contraindications,^[13]^ increasing interest in the use of non-pharmacological treatments for minimizing dementia progression. More commonly considered non-pharmacotherapies (NPTs) include cognitive stimulation, cognitive rehabilitation, music therapy, computerized cognitive training, exercise and noninvasive brain stimulation, which have been associated with improved cognitive function and related behaviors such as decreased agitation and depressed mood.^[14–19]^

Currently, no disease-modifying treatments have proven fully effective in controlling the symptoms of AD. Despite focused research efforts on preclinical and early stage AD to develop new disease-modifying treatments that reduce the pathophysiological changes associated with AD^[20]^ (i.e., amyloid deposition, phosphorylation and protein aggregation), no significant clinical effect have been achieved, to date.^[21]^ Caring for people with AD and dementia requires multidisciplinary health care team involvement (e.g., psychologists, occupational therapists, etc) and caregivers to deliver a comprehensive and individualized treatment plan. NPTs tend to be less invasive, safe and with fewer side effects with a goal of maintaining cognitive function for as long as possible, thus reducing disability, maintaining independence longer and improving patients and caregivers’ quality of life (QoL).

Despite increasing evidence of the benefits associated with PTs and NPTs, comparisons between both have been limited. In addition, implementation of real-world evidence remains challenging given the financial and resource limitations in most healthcare settings. As such, this study will provide an overview and efficacy comparisons between individual PTs and NPTs for the treatment of AD characterized by cognitive, function status, behaviors, and QoL for patients. We will also consider caregivers stress and burden as secondary outcomes. Network meta-analyses will be applied to update and estimate treatment effects for all regimens, ranking each against the outcomes indicated. The findings from this systematic review will provide a platform for future research design and cost effectiveness comparisons of pharmacological and non-pharmacological dementia treatments in real clinical practice, particularly in resources limited settings.

## 2. Methods and design

This NMA will be conducted in accordance with PRISMA guidelines for network meta-analyses and the protocol was registered with the International Prospective Register of Systematic Reviews (PROSPERO) in February 2021 (registration number CRD42021228245).

### 2.1. Information sources and search strategy

Relevant systematic reviews and meta-analyses will be identified by a single reviewer (CB) from PubMed and Scopus from initiation to June 2021. The search terms and strategies follow the PICOs guidelines of patients (P), interventions/ comparators (I/C), outcomes (O), and study design (S) as follows:

P: “Alzheimer dementia; I/C: donepezil, rivastigmine, galantamine, memantine, huperzine-A, EGb761, Yokukansan (TJ-54), “cognitive stimulation therapy,” “cognitive rehabilitation,” “music therapy,” “physical activity,” “noninvasive brain stimulation,” “non-pharmacological therapy”; O: cognitive, behaviors, function, “quality of life”; S: meta-analysis.

Searches will be updated every 3 months until October 2022.

### 2.2. Study selection process

#### 2.2.1. Type of study to be included.

Systematic reviews of randomized controlled trials (RCTs) will be selected that meet the following criteria:

□ Conducted in elderly patients aged 60 years or older with AD
□ Reported PMA or NMA for pooled relative treatment effects for at least PTs or NPTs treatment pairs
□ Provided at least 1 of the following outcomes for patients/caregivers: cognitive function status, rate of falls, behavior, and QoL.

Individual RCTs included in selected systematic reviews will be re-selected based on the following criteria:

□ Undertaken in elderly patients aged 60 years or older with AD
□ Compared relative treatment effect of PTs to placebo or other PTs or NPTs to usual care or to other NPTs.
□ Provided at least 1 of the following outcomes for patients/caregivers: cognitive function status, rate of falls, behavior, QoL, and caregiver stress or burden.

**Figure 1.**
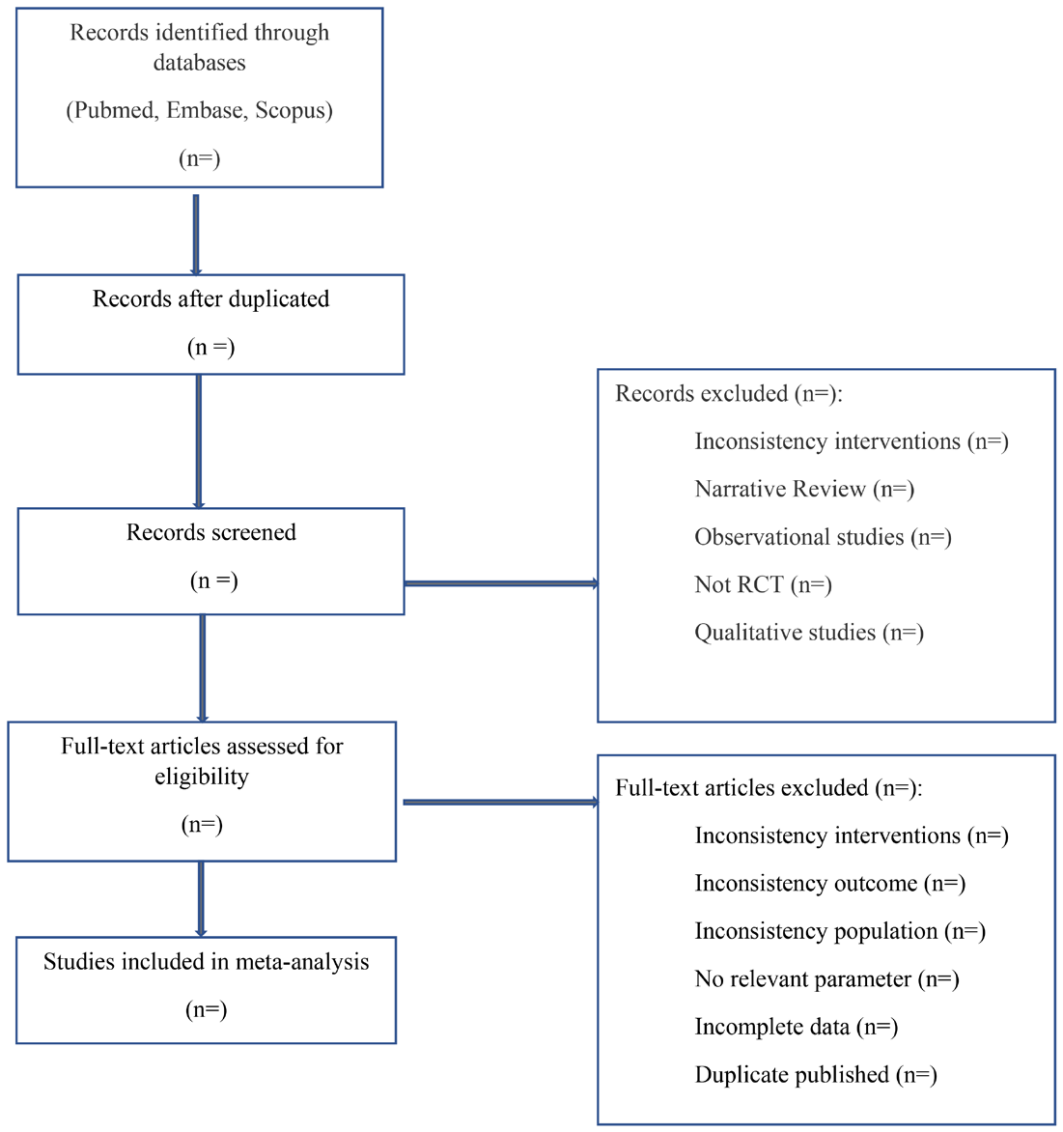
Flow diagram of study selection process.

Two groups of reviewers will independently select studies by screening the titles and abstract of identified articles. Full articles will be retrieved if decision cannot be made. Data will be validated and assessed if there is any discrepancy. Disagreement will be solved by discussion and consensus with the third person. Individual RCTs will be excluded where insufficient data is available for pooling following 3 attempted contacts with the corresponding author. The process of study selection is shown in Figure 1.

#### 2.2.2. Population.

We will include any RCT that comprised elderly participants aged 60 years or older diagnosed with AD using their national standard AD diagnostic criteria, such as the Diagnostic and Statistical Manual of Mental Disorders (DSM) IV^[22]^ or DSM-5,^[23]^ the National Institute of Neurological and Communicative Disorders and Stroke-Alzheimer Disease and Related Disorders Association,^[24]^ and/or National Institute on Aging (NIA) and the Alzheimer Association criteria.^[24]^ We will use the Mini-Mental State Examination (MMSE) or Montreal Cognitive Assessment/Clinical Dementia Rating Scale for additional screening.

#### 2.2.3. Intervention.

Interventions of interest include PTs and NPTs for AD, example of which are outlined in Table 1.

**Table 1:** Examples of pharmacological and non-pharmacological interventions for Alzheimer dementia (AD).

*Pharmacological interventions*
Acetylcholinesterase inhibiter (AchEIs):
Donepezil: standard dose (5–10 mg), high dose (23 mg)
Galantamine: standard dose (8–24 mg)
Rivastigmine: standard dose (6–12 mg capsule, 9.5 mg patch), high dose (13.6 mg patch)
Memantine: NMDA receptor antagonist standard dose (10–20 mg)
Herbs;
Huperzine: huperzine serrata
Egb 761: Gingo biloba extract
Yokukansan (TJ-54)
Ginseng: Korea red ginseng, Panax Ginseng
Chinese herbs: Ba wei Di Huan wan
Cooked herbs: Saffron (spice), Mellisa officialnalis (lemon balm), salvia officialnalis (sage)
*Non-pharmacological interventions*
Cognitive stimulation therapy
Cognitive rehabilitation
Cognitive therapy consisting of a structured program of a standard set of tasks designed to address various cognitive functions such as memory, attention, language, or executive function
Physical activity or exercise, e.g., Aerobic only, Non-aerobic, Combination or multicomponent, High intensity functional exercise
Occupational therapy
Caregiver education
Alternative therapy e.g., Massage, Recreation therapy, Aromatherapy, Light therapy, Art therapy, Therapeutic touch, Acupuncture, environment home modification
Music therapy
Psychosocial intervention e.g. psychological therapy, social intervention
Multicomponent therapy combining cognitive stimulation, exercise, and group education.
Multisensory behavior therapy
Noninvasive brain stimulation: rTMS, and tDCS
Pet therapy

#### 2.2.4. Comparators.

Eligible within study comparator groups will include usual care or placebo or another PT or NPT for AD treatment option.

#### 2.2.5. Outcomes.

Outcomes of interest include cognitive function, behavioral and psychological symptoms of dementia (BPSD), QoL, functional status and rate of falls. Cognitive function assessment within the original studies commonly use the MMSE, Alzheimer Disease Assessment-Scale Cognitive (ADAS-Cog), Global Cognitive Functions (e.g., Clock drawing test), Clinical Global Impression of Change (CGIC), Clinician’s Interview-Based Impression of Change (CIBIC), self or caregiver reported functions (caregiver input), Clinical Dementia Rating (CDR). BPSD are commonly evaluated by neuropsychiatric inventory (NPI), brief psychiatric rating scales and other behavior assessment tools. Quality of life (QoL) and functional status are measured by Quality of life in AD (QoL-AD), Alzheimer Disease Cooperative Study Activity of Daily Living (ADCS-ADL), interviews for deterioration in daily living activities in Dementia (IDDD), Barthel or modified Barthel index. Rate of falls is defined as the number of reported falls or number of participants falling. A fall was defined as “a person unintentionally coming to rest on the ground, floor or other level.”^[25]^

Secondary outcomes include caregiver stress or burden measured by the caregiver stress scale (CSS), and Zarit burden inventory scale (ZBI) respectively. Other potential secondary outcomes of treatment safety will include reports of serious adverse events such as fractures, falls, stroke or death.

### 2.3. Data extraction

Prior to data extraction, a charting exercise will be undertaken to better inform the data extraction process form: types of studies retrieved, outcomes reported, and effect measures recorded for each study. All systematic review data will be extracted independently by 2 groups of reviewers. Disagreements will be resolved by a third party. Systematic review data will include systematic review characteristics (pairwise or network meta-analysis), number of RCTs included, number of interventions/comparators, type of outcomes, methods used for pooling relative treatment effects (standardized/unstandardized mean difference (SMD/UMD) for continuous outcomes; odd ratios (ORs), and relative risks (RRs) for dichotomous outcomes). The following data will be extracted from individual RCTs: patient characteristics (such as age, percentage female, body mass index, severity of dementia, duration of illness, and co-morbidities, including diabetes, hypertension, cardiovascular disease status, etc), intervention/comparator, type of outcomes and tool/scale used to determine outcome measures.

In addition, outcomes of efficacy will be extracted for short-term (≤24 weeks), and long-term (>24 weeks) follow-up where interventions have been assessed at multiple time-points. Extracted data for pooling will include the number of patients, mean and standard deviation (SD) by intervention group for continuous outcomes, contingency data of intervention and dichotomous outcomes.

### 2.4. Risk of bias and quality assessment

Risk of bias will be assessed using the Risk of Bias in Systematic Reviews (ROBIS) checklist^[26]^ by 2 independent reviewers, disagreement will be resolved by third party. Four domains will be considered including study eligibility criteria, methods used to identify and/or select studies, data collection and appraisal of studies, and synthesis/findings. The result will be graded as low or high risk of bias if there is sufficient information to assess, otherwise, it will be graded as unclear.

Risk of bias of individual RCTs will be assessed using the version 2 of the Cochrane risk-of-bias tool for randomized trials (RoB 2) considering the 5 domains of randomization, deviation from the intended intervention, missing outcome data, outcome measurements, and selection of the reported results. Each domain will be rated as low, some concerns, and high risk according to the risk-of-bias tool algorithm. RCTs will be considered as high risk of bias if at least 1 domain is rated high risk; low risk of bias if all domains are rated as low risk; otherwise, the RCT will be adjudged to have some concerns. Any disagreement will be resolved by consensus or resolved by third party.

### 2.5. Data synthesis

A 2-stage NMA will be performed as follows: Relative treatment effects (e.g., SMD, UMD, ln ORs/ln RR) and variance estimates will be estimated for individual RCTs and pooled across RCTs using a consistency model. All possible relative treatment comparisons will then be estimated accordingly. Consistency assumptions will be assessed using design-treatment interaction methods with a loop-specific approach to identify a loop that causes inconsistencies, where present. Patient characteristics associated with specific loops will be explored. Treatment efficacy will be ranked for each outcome using surface under cumulative ranking curves (SUCRA). Comparison adjusted funnel plots will assess publication bias.

Subgroup analysis will be performed to explore source/s of heterogeneity and inconsistency if data are available and sufficient for performing. Potential factors include age < 85 years versus 85 years or older, stage of disease as defined by the functional assessment staging test (FAST) criteria,^[27]^ etc. In addition, a sensitivity analysis will be performed by excluding studies with high risk of bias, very old age, low/high effect size/variance (e.g., 25th or 75th percentile) to determine the robustness of the findings reported. Furthermore, treatment ranking by SURCA of all outcomes will be plotted to identify which treatments gain beneficial on most outcomes. All analyses will be stratified by time at outcome assessment (i.e., short term, and long term), and performed using STATA 17.

## 3. Discussion

Currently, a comprehensive review to compare pharmacological and non-pharmacological treatments to determine the most efficacious and cost-effective options for AD patients is lacking. Despite evidence supporting small to medium range benefits associated with pharmacological treatments, many dementia patients find themselves with limited treatment options due to costs or the tolerability of some medications. In addition, non-pharmacological treatments require multidisciplinary specialized teams which may be limited in real world clinical practice, particularly in resource limited settings. This systematic review and NMA will inform the knowledge gap with findings to guide evidence-based decision-making in AD treatment strategies to support recommended AD management, not only for patients but also for family caregivers.

## Acknowledgments

We are grateful to Associate Professor Thunyarat Anothaisintawee for providing scientific suggestion and direction of this manuscript. Also, we would like to thank Miss Paichit Inpanya for coordinating the project management.

## Author contributions

**Conceptualization:** Ammarin Thakkinstian, Chitima Boongird, Worapong Tearneukit.

**Funding acquisition:** Chitima Boongird.

**Investigation:** Chitima Boongird, Wannisa Wongpipathpong.

**Methodology:** Ammarin Thakkinstian, Gareth J McKay.

**Writing – original draft:** Chitima Boongird.

**Writing – review & editing:** Ammarin Thakkinstian, Gareth J McKay, Wannisa Wongpipathpong, Worapong Tearneukit.
